# Logistic Regression of Ligands of Chemotaxis Receptors Offers Clues about Their Recognition by Bacteria

**DOI:** 10.3389/fbioe.2017.00088

**Published:** 2018-01-22

**Authors:** Takashi Sagawa, Ryota Mashiko, Yusuke Yokota, Yasushi Naruse, Masato Okada, Hiroaki Kojima

**Affiliations:** ^1^National Institute of Information and Communications Technology (NICT), Advanced ICT Research Institute, Kobe, Japan; ^2^Department of Bioengineering, Nagaoka University of Technology, Nagaoka, Japan; ^3^Department of Complexity Science and Engineering, The University of Tokyo, Kashiwa, Japan

**Keywords:** bacterial chemotaxis, chemotaxis receptor, machine-learning, QSAR, sparse modeling, logistic regression

## Abstract

Because of relative simplicity of signal transduction pathway, bacterial chemotaxis sensory systems have been expected to be applied to biosensor. Tar and Tsr receptors mediate chemotaxis of *Escherichia coli* and have been studied extensively as models of chemoreception by bacterial two-transmembrane receptors. Such studies are typically conducted using two canonical ligands: l-aspartate for Tar and l-serine for Tsr. However, Tar and Tsr also recognize various analogs of aspartate and serine; it remains unknown whether the mechanism by which the canonical ligands are recognized is also common to the analogs. Moreover, in terms of engineering, it is important to know a single species of receptor can recognize various ligands to utilize bacterial receptor as the sensor for wide range of substances. To answer these questions, we tried to extract the features that are common to the recognition of the different analogs by constructing classification models based on machine-learning. We computed 20 physicochemical parameters for each of 38 well-known attractants that act as chemoreception ligands, and 15 known non-attractants. The classification models were generated by utilizing one or more of the seven physicochemical properties as descriptors. From the classification models, we identified the most effective physicochemical parameter for classification: the minimum electron potential. This descriptor that occurred repeatedly in classification models with the highest accuracies, This descriptor used alone could accurately classify 42/53 of compounds. Among the 11 misclassified compounds, eight contained two carboxyl groups, which is analogous to the structure of characteristic of aspartate analog. When considered separately, 16 of the 17 aspartate analogs could be classified accurately based on the distance between their two carboxyl groups. As shown in these results, we succeed to predict the ligands for bacterial chemoreceptors using only a few descriptors; single descriptor for single receptor. This result might be due to the relatively simple topology of bacterial two-transmembrane receptors compared to the G-protein-coupled receptors of seven-transmembrane receptors. Moreover, this distance between carboxyl groups correlated with the receptor binding affinity of the aspartate analogs. In view of this correlation, we propose a common mechanism underlying ligand recognition by Tar of compounds with two carboxyl groups.

## Introduction

**Figure 1:**
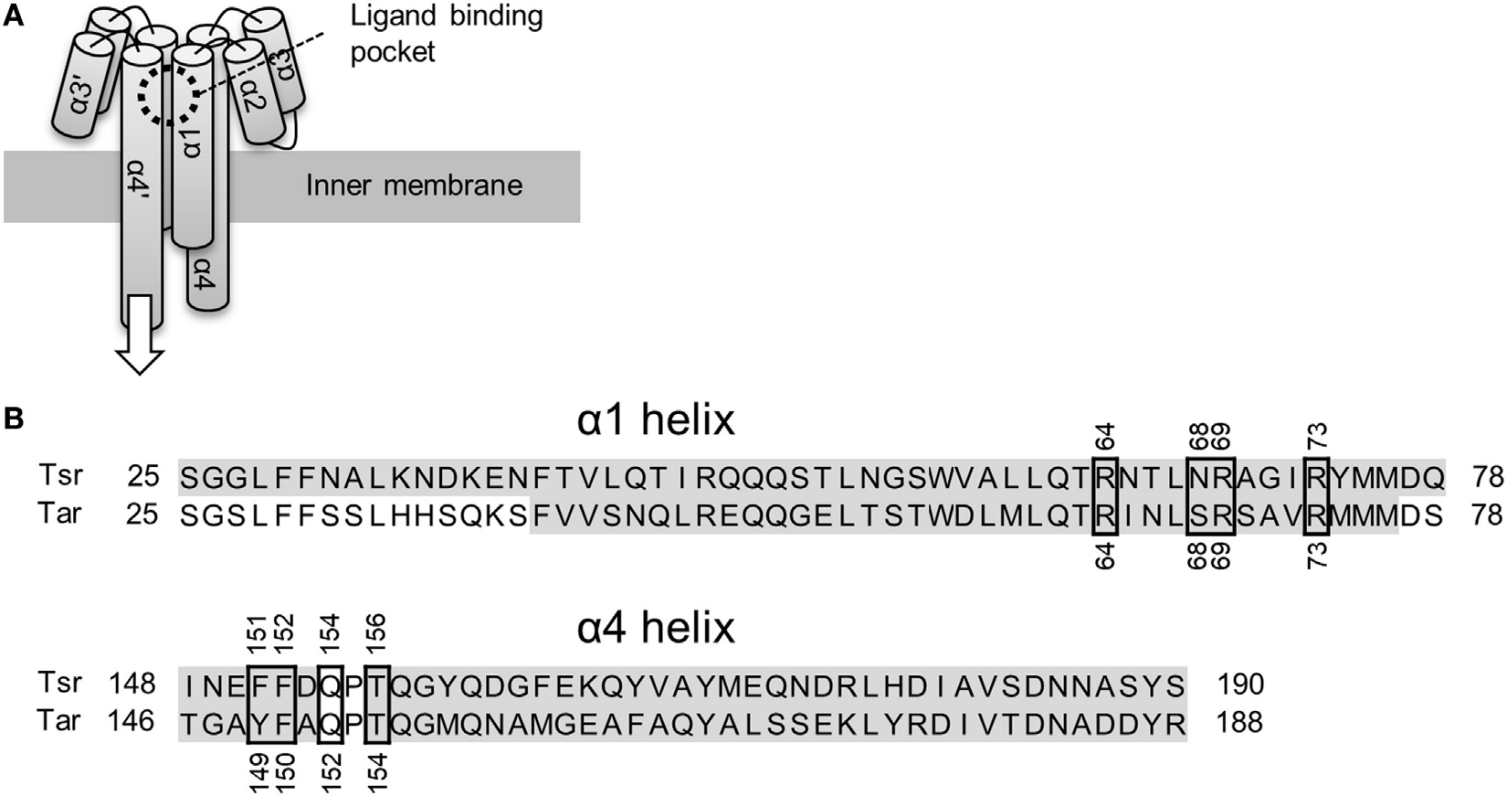
Schematics of the ligand binding domain of chemoreceptor Tar and Tsr. **(A)** Tar and Tsr consist of four α helices, α1, α2, α3, and α4. The α1 and α4 helices span the inner membrane of the *Escherichia coli* cells. The ligand binding pocket is composed of the α1 and α4 helices. The binding of the ligand causes a piston-like displacement of the α4 helices transmitting a biochemical signal into the cytoplasm. The white arrow shows the direction of the piston-like displacement of the α4 helices caused by the ligand binding. **(B)** Amino-acid sequence alignment of the α1 and α4 helices. Accession number of Tar and Tsr were P07017 and P02942, respectively. These sequences were aligned with Clustal Omega (http://www.ebi.ac.uk/Tools/msa/clustalo/). The residues highlighted in gay constitute the α-helical regions. The α4 helix in turn consists of two shorter α helices: α4a and α4b. The ligand binding residues are highlighted by black, square boxes. The numbers around the black squares mark the sequence portion of the respective residues.

Bacterial cells swim toward favorable directions by sensing environmental signals through their chemotaxis receptors (Wadhams and Armitage, 2004). The *Escherichia coli* receptors Tsr and Tar have been extensively studied as models for bacterial chemoreceptors. Tsr and Tar are two-transmembrane receptors whose ligand binding domain consists of four α helices (Figure 1A). The chemoreceptors are homodimeric in nature and their ligand binding pocket is composed of opposite pairs of α1 and α4 helices contributed by each monomer subunit. Binding of the ligand to the pocket is thought to induce a piston-like displacement of the membrane-spanning signaling-helix α4 (Falke and Erbse, 2009). Thus, the displacement transmits a signal into the cytoplasm and culminates in a change in the swimming behavior of the bacterium (for reviews refer Sourjik, 2004; Wadhams and Armitage, 2004; Krell et al., 2011).

The mechanism of ligand recognition by Tar and Tsr has been studied extensively by utilizing structural (Milburn et al., 1991; Scott et al., 1993; Yeh et al., 1993, 1996; Bowie et al., 1995; Tajima et al., 2011; Mise, 2016), genetic (Wolff and Parkinson, 1988; Lee and Imae, 1990; Mowbray and Koshland, 1990; Tajima et al., 2011), and computational approaches (Bi et al., 2013). These reports usually encompassed recognition of canonical ligands: l-aspartate for Tar and l-serine for Tsr. Direct interaction between the ligands and the ligand binding pockets has been already revealed by structural studies; residues essential for ligand recognition have also been described (Tajima et al., 2011; Figure 1B.). In addition to the canonical ligands, various analogs of them are known to act as ligands for Tar and Tsr (Mesibov and Adler, 1972; Hedblom and Adler, 1983). However, while the detailed mechanism of the recognition of the canonical ligands is known, the mechanisms underlying the recognition of the analogs remain unstudied. The ligand recognition of canonical ligands for Tar and Tsr were performed by only eight residues in the ligand binding pockets (Figure 1B). In terms of molecular recognition mechanism, how these residues recognize these diverse analogs is one of great interest. In terms of engineering, it is important to know a single species of receptor can recognize various ligands to utilize bacterial receptor as the sensor for wide range of substances (Derr et al., 2006; Bi et al., 2013, 2016; Bi and Lai, 2015).

To deduce the features of recognition common to the analogs, we distinguished the selective binding of ligands using machine-learning. Sets of previously known 38 attractants that act as ligands for the chemoreceptors, and 15 non-attractants were classified using logistic regression. The physicochemical properties of these compounds were computed from their molecular structures (Eguchi et al., 2015). This method is known as the quantitative structure–activity relationship (QASR) method and it is usually employed in drug discovery or toxicity studies. Using the strategy of exhaustive-search (ES) methods (Igarashi et al., 2016), the minimum electron potential of the compounds was identified as the most effective descriptor, which was common to all classification models. This descriptor by itself could classify attractants and non-attractants with 79% accuracy (42/53). Eight of the eleven cases of misclassification carried two carboxyl groups, which means they were analogs of aspartate. When considered separately, the aspartate analogs (17 cases) were classified by considering the distance between the two carboxyl groups (16/17). As shown in these results, we succeed to predict the ligands for bacterial chemoreceptors using only a few descriptors; single descriptor for single receptor. This result might be due to the relatively simple topology of bacterial two-transmembrane receptors compared to the G-protein-coupled receptors (GPCRs) of seven-transmembrane receptors. Moreover, the binding affinity of these aspartate analogs showed a correlation with the distance between their carboxyl groups. From this result, we proposed a mechanism common to ligand recognition by chemotaxis receptors of *E. coli*.

## Materials and Methods

### Selection of Sample Sets

Attractants and non-attractants were selected from Mesibov and Adler, 1972, Table 6, in which the response of wild-type *E. coli* (AW518) to several compounds was described, as measured by the capillary assay. The table listed 53 compounds including l-aspartate, l-serine, and their analogs (Table S1 in Supplementary Material). Among these 53 compounds, 52 compounds were selected, except for glutathione. The molecular weight of the glutathione (*M*_W_ = 307.3 Da) is too large for it to fit into the ligand binding pocket of Tar Da (Wei et al., 2010; Bi et al., 2013). In addition, l-glutamate was included into our selection as an aspartate analog from Table 3 of the same report (Mesibov and Adler, 1972). These 53 compounds contained 38 attractants and 15 non-attractants. Each of the 38 attractants was accompanied by a parameter describing the concentration required to induce the cellular response of wild-type *E. coli* (*K*_D_, M).

### Calculation of Physicochemical Properties

The stable structure of the compound was determined by quantum chemical calculation with the PM6 semi empirical method contained in the Spartan ′14 suite (Wavefunction, Inc., California). From the stable structure of compounds, 8 molecular properties and 12 QSAR descriptors were obtained as descriptors (Eguchi et al., 2015). Molecular properties were as followed: formation energy (*E*, kJ/mol), formation energy in water (*E*_aq_, kJ/mol), solvation energy (*E*_sol_ = *E*_aq_ – *E*, kJ/mol), molecular weight (*M*_W_, Da), energy of the highest occupied molecular orbital (HOMO; *E*_H_, eV), and energy of the lowest unoccupied molecular orbital (LUMO; *E*_L_, eV), HOMO–LUMO gap (*E*_H_ − *E*_L_, eV), and total dipole moment (*D*, debye). QSAR descriptors were as followed: area of space-filling model (*A*_CPK_, Å^2^), polar surface area (*PSA*, Å^2^), volume of space-filling model (*V*_CPK_, Å^3^), ovality of space-filling model (*O*_CPK_), accessible area (*AA*, Å^2^), polar area (*PA*, Å^2^), minimum electron potential (*q*^−^, kJ/mol), accessible polar area (*APA*, Å^2^), minimum local ionization potential (*q*^ion−^, kJ/mol), maximum electron potential (*q*^+^, kJ/mol), octanol–water partition coefficients (Log*P*), and polarizability (*P*).

### Development of Classification Models

Models for classifying the ligands of *E. coli* chemoreceptors were constructed using the scikit-learn machine learning module (Pedregosa et al., 2011) and XGBoost (Chen and Guestrin, 2016). To build a classification model, the attractants and non-attractants were assigned the dependent variable (*y*) values of 1 and −1, respectively. The 10 physicochemical parameters described above were input as independent variables. Before the classification, all descriptor values were normalized using following equation:

$$z=\frac{x-\mu }{\sigma }$$

where μ and σ are mean and SD of the descriptor value.

### Estimation of the Effective Size

Cohen’s effective size *d* of each descriptor was calculated with following equation:

$$d=\frac{\left|\mu_{1}-\mu_{2}\right|}{s}$$

$$s=\sqrt{\frac{(n_{1}-1)\sigma_{1}^{2}+(n_{2}-1)\sigma_{2}^{2}}{n_{1}+n_{2}-2}}$$

where μ, σ, and *n* denote the mean value of the descriptors, the SD of the descriptors, and number of attractant or non-attractant compounds, respectively. By convention, *d* ~ 0.2 is considered a small effect, *d* ~ 0.5 is considered a medium effect, and *d* ~ 0.8 is considered a large effect.

## Results

### Determination of the Most Effective Descriptor for Classification

**Figure 2:**
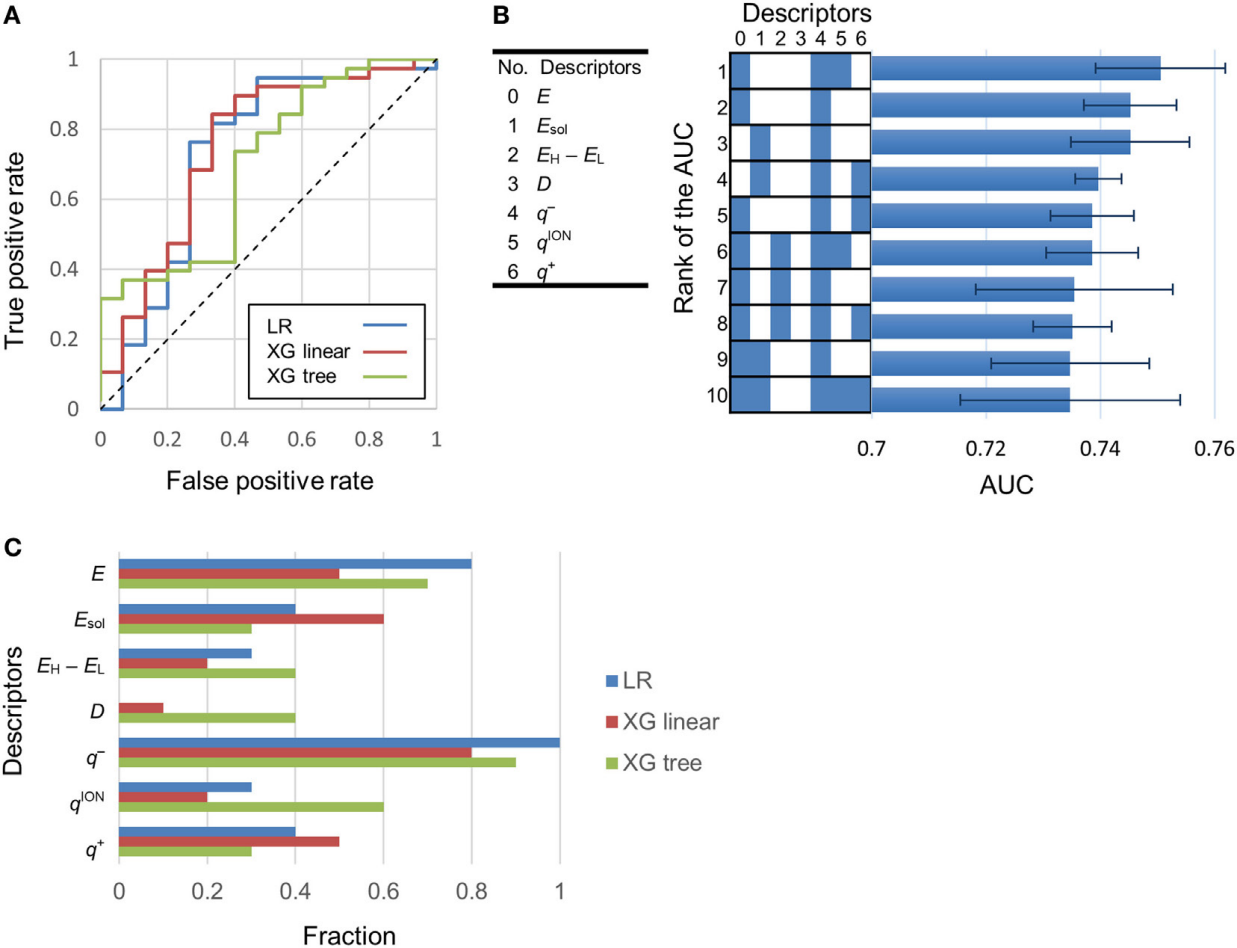
Determination of the most effective descriptor for classification. **(A)** Typical receiver operating characteristic (ROC) curves of highest area under the curve (AUC) models. LR, XG linear, and XG tree showed logistic regression, XGBoost (linear model) and XGBoost (tree model). AUC of these ROC curves were 0.73, 0.75, and 0.7, respectively. **(B)** Left-hand side: serial numbers of the descriptors. Right-hand side: AUC of the top 10 classification models. Descriptors included in a model are labeled blue. *Error bars* in the right-hand side graph show the SD of the AUC. **(C)** Fraction of models in which a descriptor is present. A fraction value of 1.0 means that the descriptor was present in all of the top 10 classification models.

Using logistic regression and eXtreme Gradient Boosting (XGboost) (Chen and Guestrin, 2016), we developed classification models to categorize various compounds as attractants or non-attractants. These compounds included l-aspartate, l-serine, and their analogs. To provide inputs for the classification models, we derived 20 physicochemical properties of each compound from its molecular structure using quantum chemical calculation (see Materials and Methods). The physicochemical properties are summarized in Table S1 in Supplementary Material. From the 20 physicochemical properties, we removed 13 redundant variables (*E*_aq_, *M*_W_, *E*_H_, *E*_L_, *A*_CPK_, PSA, *V*_CPK_, *O*_CPK_, AA, PA, APA, Log*P*, and *P*) presenting correlation coefficients greater than 0.7 using Spearman-ranked correlation coefficient values (Table 1). The remaining seven variables (*E, E*_sol_, *E*_H_ − *E*_L_, *D, q*^−^, *q*^ion−^, and *q*^+^) were selected for model construction. The classification models were constructed by choosing one or more of the physicochemical properties at a time. (Thus, we obtained Σ*_n_C_k_* = 127 models: *n* physicochemical properties chosen *k* at a time.) This strategy is termed as the ES method (Igarashi et al., 2016). These models were optimized by employing 10-fold cross-validation. From the optimized models, receiver operating characteristic (ROC) curves were derived to quantify the area under the curve (AUC) which is an appropriate measure for describing model performance (Figure 2A). Calculation of the AUC was performed five times in each optimized model. The maxim averaged AUC of each classification methods was as follows; logistic regression: 0.75 ± 0.01, XGBoost (linear model): 0.75 ± 0.01, and XGBoost (tree model): 0.73 ± 0.03 (mean ± SD, *n* = 5). The AUC of the top 10 classification models were ranged between 0.73 and 0.75 (logistic regression); between 0.74 and 0.75 [XGBoost (linear model)]; between 0.70 and 0.73 [XGBoost (tree model)] (Figure 2B). These AUC values exceeded the 0.7 showing fair performance of these classifiers. In these top 10 classification models, averaged value of the accuracy in the classification was logistic regression: 80.8%, XGBoost (linear): 78.3%, and XGBoost (tree): 75.3%.

**Table 1:** Correlation analysis of each descriptors.

	*E*	*E*_aq_	*E*_sol_	*M*_W_	*E*_H_	*E*_L_	*E*_H_ − *E*_L_	*D*	*A*_CPK_	PSA	*V*_CPK_	*O*_CPK_	AA	PA	*q^**−**^*	APA	*q*^ion^*^**−**^*	*q^**+**^*	Log*P*	*P*
*E*	1.00																			
***E***_aq_	**0.99**	1.00																		
*E*_sol_	0.56	0.64	1.00																	
***M*_W_**	**−0.77**	**−0.81**	**−0.73**	1.00																
***E*_H_**	0.48	0.43	−0.01	−0.06	1.00															
***E*_L_**	0.68	0.68	0.43	−0.47	0.46	1.00														
*E*_H_ − *E*_L_	−0.26	−0.29	−0.26	0.33	0.22	−0.61	1.00													
*D*	−0.14	−0.17	−0.54	0.41	0.23	−0.13	0.18	1.00												
***A*_CPK_**	−0.62	−0.66	−0.63	**0.95**	0.11	−0.29	0.34	0.39	1.00											
**PSA**	**−0.70**	**−0.75**	**−0.86**	**0.82**	−0.16	−0.48	0.25	0.42	**0.71**	1.00										
***V*_CPK_**	−0.62	−0.66	−0.63	**0.95**	0.11	−0.30	0.34	0.39	**1.00**	**0.70**	1.00									
***O*_CPK_**	−0.58	−0.62	−0.61	**0.92**	0.11	−0.25	0.31	0.34	**0.98**	**0.71**	**0.97**	1.00								
**AA**	−0.50	−0.54	−0.55	**0.88**	0.22	−0.23	0.40	0.37	**0.97**	0.61	**0.97**	**0.95**	1.00							
**PA**	−0.69	**−0.74**	**−0.81**	**0.87**	−0.15	−0.50	0.28	0.49	**0.80**	**0.83**	**0.80**	**0.77**	**0.71**	1.00						
*q*−	−0.49	−0.46	−0.04	0.16	**−0.76**	−0.54	0.02	−0.25	0.00	0.17	0.01	−0.03	−0.07	0.19	1.00					
**APA**	−0.66	**−0.71**	**−0.79**	**0.81**	−0.19	−0.55	0.34	0.49	**0.73**	**0.80**	**0.73**	0.69	0.66	**0.97**	0.28	1.00				
*q*^ion^−	−0.23	−0.21	0.04	0.14	−0.27	−0.17	0.16	−0.09	0.18	0.12	0.17	0.15	0.19	0.10	0.19	0.16	1.00			
*q*^+^	−0.59	−0.60	−0.49	0.42	−0.28	**−0.84**	0.50	0.13	0.25	0.47	0.25	0.21	0.19	0.41	0.47	0.45	−0.05	1.00		
**Log*P***	0.12	0.19	**0.74**	−0.42	−0.19	0.05	0.03	−0.62	−0.36	−0.65	−0.36	−0.40	−0.32	−0.53	0.28	−0.48	0.32	−0.18	1.00	
***P***	−0.62	−0.66	−0.63	**0.95**	0.12	−0.32	0.39	0.39	**0.99**	**0.70**	**1.00**	**0.96**	**0.97**	**0.79**	0.02	**0.73**	0.17	0.27	−0.34	1.00

*Descriptors showing high correlation coefficient (>0.7) were subjected to removal. Both the removed descriptors and high correlation coefficient (>0.7) were bolded*.

To isolate the most effective descriptor for obtaining accurate classification models, the frequency of each descriptor in the top 10 classification models was tallied (Figure 2C). The minimum electron potential present on the compound surface (*q*^−^) was a descriptor that was present in most of the 10 classification models. The effectiveness of the descriptors in generating accurate classification models was also assessed by comparing the average value of a descriptor among attractants with its corresponding average value among non-attractants (Table 2). Application of *t*-tests showed that the average values of *E*_sol_, *D*, and *q*^−^ were significantly different between attractants and non-attractants (*p* = 0.02, *p* = 0.009, *p* = 0.03, respectively). The descriptor *q*^−^ showed the smallest *p*-value, and this result complements the inclusion of *q*^−^ in the top 10 classification models. On the other hand, despite showing a significant difference between attractants and non-attractants, the dipole moment (*D*) descriptor was not included in most of the top 10 classification models. This discrepancy could be because, irrespective of their group average, several attractants do not show a dipole moment because of their symmetric structures (fumarate and succinate). The effectiveness of the descriptors was assessed again using the measure of effect size called Cohen’s *d* (Cohen, 1988). It was calculated as the difference mean values of the two groups of compounds normalized by their combined SD (see Materials and Methods for the full formula). The descriptor *q*^−^ showed the largest effect size of 0.96. These results corroborate that *q*^−^ was the most effective descriptor to classify compounds into attractants and non-attractants. The average values of *q*^−^ among attractants and non-attractants were 300 ± 31 kJ/mol (*n* = 38) and –269 ± 36 (*n* = 15) kJ/mol (mean ± SD), respectively. Therefore, the *q*^–^ of attractants tended to be smaller than that of non-attractants.

**Table 2:** Averaged value of descriptors in attractants and non-attractants.

Descriptor	Attractant (*n* = 38)	Non-attractant (*n* = 15)	*p*^a^	*d*^b^
*E* (kJ/mol)	−596 ± 233	−566 ± 186	0.7	0.14
*E*_sol_ (kJ/mol)	−74 ± 18	**−**61 ± 16	0.02	0.76
*E*_H_ − *E*_L_ (eV)	−10.6 ± 1.0	−10.4 ± 1.0	0.7	0.13
*D* (debye)	2.5 ± 1.1	1.8 ± 0.7	0.009	0.67
***q*^−^ (kJ/mol)**	**−300 ± 31**	**−269 ± 36**	**0.003**	**0.96**
*q*^ION^ (kJ/mol)	50.3± 6.4	51.7 ± 4.5	0.5	0.23
*q*^+^ (kJ/mol)	196± 31	198 ± 13	0.8	0.05

*^a^p-Values were calculated from t-test*.

*^b^Cohen’s *d*, which is a measure of effect size (Cohen, 1988)*.

*The most effective descriptor is highlighted in bold*.

### Classification Using only the Minimum Electron Potential *q*^–^

**Figure 3:**
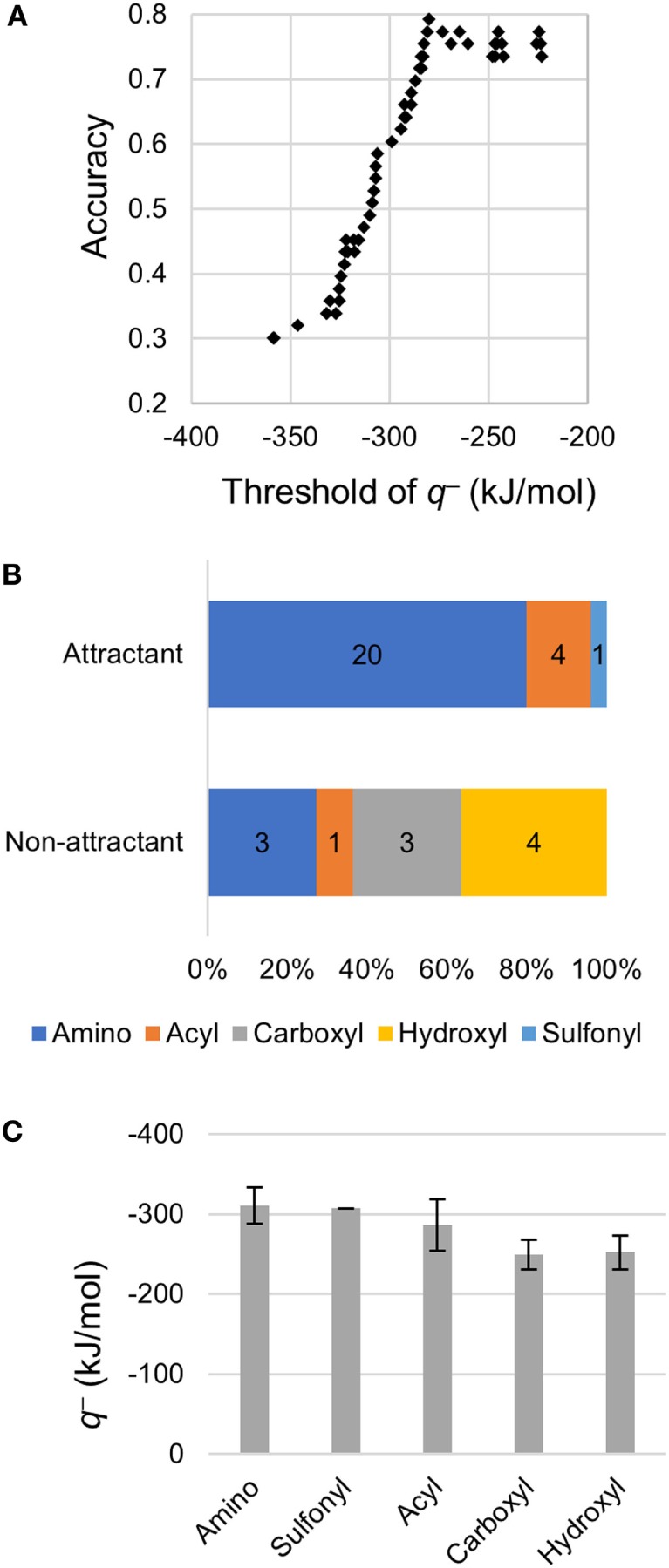
Classification using the minimum electron potential *q*^–^. **(A)** Classification using a single threshold of *q*^−^. **(B)** Distribution of *q*^−^ on functional groups of non-aspartate analogs. **(C)** Mean *q*^−^ on each functional group. Error bars show SD. Number of amino, sulfonyl, acyl, carboxyl, and hydroxyl groups observed were 31, 1, 6, 10, and 5, respectively.

Using only the most important descriptor *q*^−^, attractants and non-attractants were classified again. The classification was performed using the single threshold of the *q*^−^ (Th*_q_*_−_). If a compound had a *q*^−^ below Th*_q_*_−_ then, it was classified as an attractant, otherwise it was classified as a non-attractant (Figure 3A). When the Th*_q_*_–_ was set at –280 kJ/mol, accuracy of the classification showed maximum value, and 42/53 of the compounds were correctly classified. We emphasize: most of the attractant and non-attractant could be classified using only the minimum electron potential.

To clarify the cause of the misclassification, we analyzed the remaining 11 compounds (Table 3). Among these 11 compounds, eight showed a structure analogous to aspartate: they carried two carboxyl groups. The remaining three compounds were all analogs of serine (β-alanine, γ-amino-*n*-butyrate, and l-homocysteine). Therefore, most of the misclassification occurred on two-carboxyl compounds, which are analogous to aspartate. In a previous report, compounds with two carboxyl groups typically bind to the aspartate receptor Tar with a higher affinity than the serine receptor Tsr; the apparent dissociation constant to induce cellular response (*K*_D_) in *tsr* deletion mutant was smaller than that of *tar* deletion mutant (Mesibov and Adler, 1972). Actually, accuracy in the classification using the Th*_q_*_–_ for aspartate analogs and other than aspartate analogs were 53% (9/17) and 92% (33/36), respectively. Thus the descriptor *q*^−^ need only be used to accurately classify all ligands other than aspartate analogs, that is, serine analogs.

Then, we analyzed the *q*^−^ of non-aspartate analogs. Among 53 compounds, 17 compounds carrying two carboxyl groups were excluded because *q*^−^ did not prove to be effective for the classification of aspartate analogs. Among the non-aspartate analogs classified as attractants, most of the *q*^−^ was attributed to either amino (–NH_2_ and –NHR) or acyl groups (R-CO–) (24/25; Figure 3B). The remaining compound carried a sulfonyl group (–SO_2_). Moreover, all the 25 attractants carried the amino group. On the other hand, *q*^−^ was attributed to the carboxyl groups and hydroxyl groups on non-attractants (7/11). The mean value of *q*^−^ attributed to each functional group was quantified by analyzing all the 53 compounds (Figure 3C). The amino group showed the smallest *q*^−^, –311 ± 22 kJ/mol (mean ± SD, *n* = 31), followed by the sulfonyl –307 kJ/mol (*n* = 1) and acyl groups –287 ± 32 kJ/mol (mean ± SD, *n* = 6). On the other hand, *q*^−^ of the hydroxyl –249 ± 18 kJ/mol (mean ± SD, *n* = 10) and carboxyl groups –252 ± 21 kJ/mol (mean ± SD, *n* = 5) were higher than that of the amino, sulfonyl, and acyl groups, which are carried by the attractants. Therefore, non-aspartate attractants had smaller electron potentials. Thus, a smaller electron potential would be an essential factor for ligand recognition by Tsr.

### Descriptors for the Classification of Aspartate Analogs

**Figure 4:**
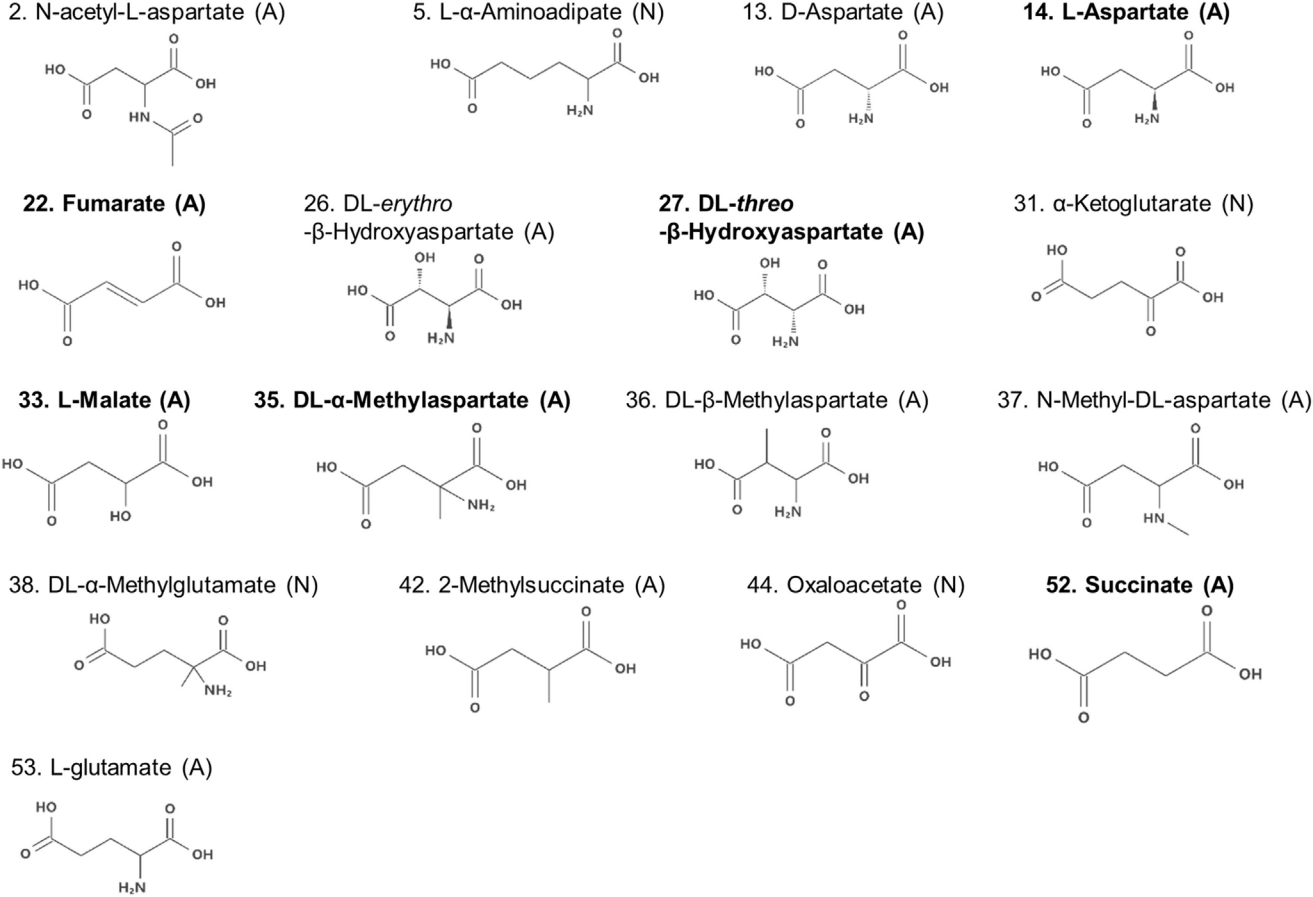
Molecular formulae of the amino acid analogs. Numbers before the name of the compound are serial numbers (Table S1 in Supplementary Material). The molecules are labeled with the brackets (A) or (N) signifying attractants and non-attractants, respectively. Compounds in bold characters bind to Tar with a higher affinity than to Tsr, as reported previously (Mesibov and Adler, 1972).

Compounds with two carboxyl groups could not classified using the descriptor *q*^−^. To find out the descriptor that was effective for classification of these aspartate analogs, their molecular structures were analyzed (Table 3; Figure 4). We focused on the number of carbon-chain atoms between the two carboxyl groups (*N*_Carbon_). Only those compounds were classified as false negatives (attractant classified as non-attractant) whose *N*_Carbon_ was the same as that of l-aspartate (*N*_Carbon_ = 2). These compounds were fumarate, dl-threo-β-hydroxyaspartate, l-malate, dl-β-methylaspartate, 2-methylsuccinate, and succinate. On the other hand, analogs whose *N*_Carbon_ was three or four were classified as false positives (non-attractant classified as attractant). These compounds were l-α-aminoadipate and dl-α-methylglutamate. Therefore, *N*_Carbon_ seemed to be an important descriptor for classifying aspartate analogs into attractants and non-attractants. Accordingly, *N*_Carbon_ was counted in all 17 aspartate analogs (13 attractants, 4 non-attractants; Figure 4; Table 4). *N*_Carbon_ of the attractants was 2, except for l-glutamate (*N*_Carbon_ = 3). On the other hand, *N*_Carbon_ of the non-attractants was more than 2 (*N*_Carbon_ = 3 or 4), except for oxaloacetate. Therefore, by assuming the *N*_Carbon_ of the attractant to be 2, attractants and non-attractants could be classified with 88.2% accuracy (15/17).

**Table 3:** Compounds misclassified according to *q*^−^.

No.	Compound	*K*_D_^a^	*N*_Carboxyl_^b^	*N*_Carbon_
4	β-Alanine		1	
5	l-α-Aminoadipate^c^		2	4
8	γ-Amino-*n*-butyrate		1	
22	Fumarate	3.E−4	2	2
24	l-Homocysteine		1	
27	dl-Threo-β-hydroxyaspartate	1.E**−**4	2	2
33	l-Malate	6.E−4	2	2
36	dl-β-Methylaspartate	3.E−4	2	2
38	dl-α-Methylglutamate^c^		2	3
42	2-Methylsuccinate	5.E−3	2	2
52	Succinate	2.E−3	2	2

*Compounds with blank values are non-attractants*.

*^a^Apparent dissociation constant calculated from the concentration of the ligand required to induce a response by wild-type *E. coli* (Mesibov and Adler, 1972)*.

*^b^Number of carboxyl groups on the compounds*.

*^c^Compounds for which the number of carbon atoms N_Carbon_ > 2*.

**Table 4:** Distance between two carboxyl-groups on l-aspartate analogs.

No.	Compounds	*K*_D_ (M)	*N*_Carbon_	^†^*N*_NH2_	*R* (Å)	Sensitivity
14	l-Aspartate	6.E−08	2	1	2.950	7.2
35	dl-α-Methylaspartate	5.E−07	2	1	2.958	6.3
36	dl-β-Methylaspartate	3.E−04	2	1	2.991	3.5
44	Oxaloacetate		2		3.002	
37	*N*-Methyl-dl-aspartate	1.E−03	2		3.006	3.0
13	d-Aspartate	1.E−05	2	1	3.084	5.0
33	l-Malate	6.E−04	2		3.151	3.2
27	dl-Threo-β-hydroxyaspartate	1.E−04	2	1	3.218	4.0
53	l-Glutamate	5.E−06	3	1	3.269	5.3
2	*N*-Acetyl-l-aspartate	1.E−03	2		3.282	3.0
22	Fumarate	3.E−04	2		3.764	3.5
42	2-Methylsuccinate	5.E−03	2		3.803	2.3
52	Succinate	2.E−04	2		3.820	3.7
26	dl-Erythro-β-hydroxyaspartate	5.E−04	2	1	3.861	3.3
38	dl-α-Methylglutamate		3	1	4.444	
31	α-Ketoglutarate		3		5.063	
5	l-α-Aminoadipate		4	1	6.358	

*The 17 compounds were arranged in ascending order of the distance between their carboxyl groups (*R*). The –NHR of the amino group was excluded from the count*.

*^a^Number of –NH_2_ groups*.

**Figure 5:**
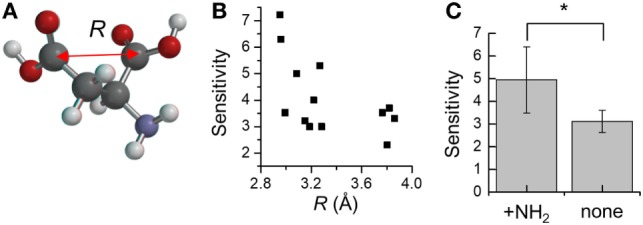
Distance between the two carboxyl-groups on aspartate analogs **(A)** Depiction of the distance between the two carboxyl groups *R*. The *R* was calculated from molecular models determined using quantum chemical calculation (see Materials and Methods). **(B)** Relation between the cellular sensitivity of compounds and their *R*. The cellular sensitivity was defined as –log_10_(*K*_D_), where *K*_D_ was the threshold of concentration required to induce a response by wild-type *Escherichia coli*. **(C)** Effect of the presence of the amino group on the sensitivity of aspartate analogs. The sensitivities of seven attractants and six non-attractants were averaged. Significant *p*-values (*p* < 0.05) are marked with a*.

Moreover, to take into account the discrepancies associated with oxaloacetate and l-glutamate, we next focused on the distance between the two carboxyl-groups in aspartate analogs. The distance between the carbon atoms of the two carboxyl groups was defined as *R* (Figure 5A). The relation between the *R* and threshold of concentration for cellular response (*K*_D_) was evaluated for the 17 aspartate analogs (Table 4). The compounds with the three largest *R* values are non-attractants, and all the other compounds are attractants, except for the oxaloacetates. Thus, the attractants can be classified in response to the distance between the carboxyl groups, using a single threshold value for *R* (*R*_Thresh_ ~ 4 Å). According to this classification, the discrepancy owing to the *N*_Carbon_ of l-glutamate (*N*_Carbon_ = 3, attractant) could be resolved. To analyze the effect of the distance between the carboxyl carbon atoms of the aspartate analogs on their binding to the receptor, the *R* and *K*_D_ were ascertained for the 13 attractants among the aspartate analogs. The cellular sensitivity of the compounds, defined as –log_10_(*K*_D_), was plotted against the *R* values (Figure 5B). An inverse correlation was observed between the sensitivity and *R* (Pearson’s correlation coefficient *r* = –0.57, *p* = 0.041).

Moreover, several of the aspartate analogs had amino groups between their two carboxyl groups. This was especially true of attractants, which have a low *K*_D_ (l-aspartate, dl-α-methylaspartate, and l-glutamate). Therefore, the effect of the –NH_2_ group on ligand binding was evaluated for the 17 aspartate analogs. Among the 13 attractants, seven compounds had single –NH_2_ groups and the remaining did not have any –NH_2_ groups. Among the four non-attractants, two had a single –NH_2_ group and the remaining did not have any –NH_2_ groups. These results show that the ratio of compounds that have an –NH_2_ group is comparable between attractants and non-attractants. Therefore, the amino group was not an essential feature of attractants of Tar. This result was consistent with the classification based on *q*^−^ which is majorly attributed to the amino group, but failed to classify the aspartate analog accurately (accuracy: 9/17). On the other hand, average sensitivities were significantly different between attractants with an amino group and those without an amino group (with –NH_2_ group: 4.9 ± 1.5, without –NH_2_ group: 3.1 ± 0.5, mean ± SD, *p* = 0.01, Figure 5C). Thus, we conclude that the –NH_2_ group was not an essential feature for classifying attractants of Tar. Instead, this group improves the binding affinity with Tar.

## Discussion

### Classification of the Ligand of Bacterial Chemoreceptors Using the QSAR Method

We classified each of the 53 chemoreceptor ligands as an attractant or non-attractant using machine-learning. To characterize these compounds, we used the QSAR method, which uses the physicochemical properties of the compounds instead of the crystal structure of the ligand binding pockets. The QSAR method can be adopted in the absence of structural information about the target, and has been applied to predict the substrate for the GPCRs (Wang et al., 2008; Michielan et al., 2009; Lounkine et al., 2010; Brogi et al., 2011; Zhang et al., 2012), which are a major target of drug discovery. In this study, we showed that the ligands of bacterial chemoreceptors could be classified into attractants or non-attractants using a combination of 20 physicochemical properties of the compounds. The classifier of the highest AUC had only 3 descriptors, which was much smaller number than previous QSAR studies for predicting substrates of various GPCRs [dopamine receptor: 98 descriptors (Zhang et al., 2012) and adenosine receptor: 300 descriptors (He et al., 2016)]. Finally, these descriptors were narrowed down to single descriptor for single chemoreceptors; *q*^−^ for Tsr and *R* for Tar. This small number of the descriptors in our model might be derived because the bacterial two-transmembrane receptors had much simpler topology than GPCR of seven-transmembrane receptors. To our knowledge, QSAR predictions have only been applied to the seven-transmembrane receptors (GPCRs). This study demonstrated for the first time that the QSAR method is applicable for predicting the ligands of the two-transmembrane receptor, and we suggested the ligand of the two-transmembrane receptors could be predicted with only a few descriptors.

### Identification of the Most Effective Descriptor Using Sparse Modeling

We succeeded in extracting a physicochemical property singly effective at classification using ES-logistic regression, which corresponds to L0 regularization of sparse modeling (Igarashi et al., 2016). The minimum electrical potential (*q*^−^) was extracted as the effective descriptor, which could classify attractants and non-attractants with 79% accuracy (42/53). Most of the false classification was observed for compounds carrying two carboxyl groups, which is analogous to aspartate (8/11). The analogs of aspartate could bind to Tar with a higher affinity. Therefore, *q*^−^ could classify most of the attractants for Tsr, but not Tar. Attractants among non-aspartate analogs mostly had their *q*^−^ attributable to the presence of amino groups. On the other hand, most of the non-attractants did not have amino groups (8/11). Therefore, the amino group might be an essential residue for ligand recognition by Tsr. The importance of the amino group for ligand recognition by Tsr has been discussed in a previous study (Tajima et al., 2011). Therefore, by combing QSAR and ES method, we succeeded in deriving clues about the ligand binding mechanism of the receptor without the information about the structure of the ligand binding pocket. We propose that the combination of the QSAR method and sparse modeling could prove to be an effective approach for understanding the mechanism of ligand recognition by receptors, the structure of whose ligand binding pocket is unresolved.

### Molecular Mechanism of Ligand Recognition by Tsr

**Figure 6:**
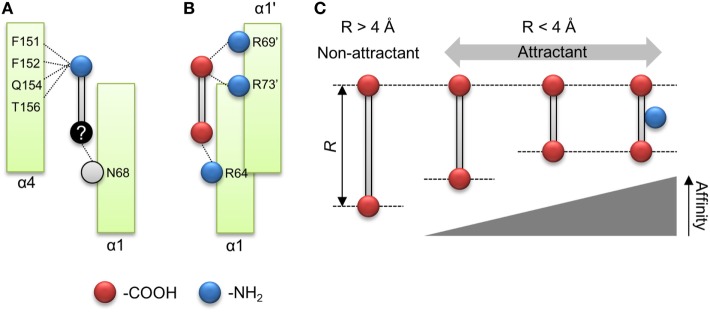
Model of ligand recognition of the aspartate analog by Tar. **(A)** Minimal model for ligand recognition by Tsr. Dotted lines indicate interactions between the compound and the ligand binding pockets. *Red* and *blue circle* shows carboxyl and amino groups. *Black circle* shows functional groups, the interaction of which is unknown. *Gray circle* shows functional groups of which interaction with the compounds was obscure. **(B)** Minimal model for ligand recognition by Tar. **(C)** Summary of rule for ligand recognition by Tar.

The importance of the amino group for ligand recognition by Tsr has been discussed in a previous report (Tajima et al., 2011). In that report, α-amino group of l-serine was shown to directly interact with following residues of the receptor α4 helix: Phe-151, Phe-152, Gln-154, and Thr-156 (Figure 1B). Therefore, the amino groups on non-aspartate analogs might also interact with these residues. In addition, the β hydroxyl group of l-serine was known to interact with the Asn-68 residue of α1 helix and the Arg-73′ of residue of the anti-parallel α1 helix. The prime denotes the residue located on the opposite homo dimer. The former was essential for ligand recognition while the latter was not. To repeat, Asn-68 was an essential residue for ligand recognition by Tsr. Thus, the residues essential for ligand recognition could be narrowed to the following: Phe-151, Phe-152, Gln-154, and Thr-156 residues on α4 helix for recognition of amino group; Asn-68 residue on α1 helix for recognition of the hydroxyl group (Figure 6A). In this model, amino and hydroxyl groups of the attractant cross-link α1 and α4 helices of the ligand-binding pocket of Tsr. However, we could not determine conclusively if the hydroxyl group was essential for ligand recognition by Tsr because several attractant serine analogs did not contain hydroxyl groups. Therefore, the mechanism of binding of compounds to Tsr remained underdetermined in this study. This study only expanded upon the importance of the amino groups in l-serine recognition and recognition of the various serine analogs. However, some limitations are worth noting about the importance of the amino groups. Despite carrying amino groups, following three compounds did not behave as attractants: β-alanine, γ-amino-*n*-butyrate, and l-homocysteine. The first two, β-alanine and γ-amino-*n*-butyrate, have –NH_2_ groups which have a small value of *q*^−^. However, it must be noted that the position of the –NH_2_ groups was different from the α-amino acid. Therefore, to explain the recognition of these compounds, other functional groups might be considered which was also essential for ligand recognition of the Tsr. Future work therefore should consider the relative position of –NH_2_ groups and other functional groups which could also act as essential residue. Further mechanistic insights involving the interaction of Asn-68 and the relative position of the –NH_2_ groups would be obtained by quantifying binding of the compound to Tsr by isothermal titration calorimetry (Tajima et al., 2011; Bi et al., 2013).

### Common Mechanism for Ligand Recognition by Tar

In contrast to the serine analogs which could be classified with physicochemical properties, the aspartate analogs could be classified using the structural properties of the compounds. The attractants for Tar could be predicted by quantifying the distance between the carboxyl groups (*R*). The attractants and non-attractants were classified by using a single *R* threshold of ~ 4 Å with 94% of accuracy (16/17). Moreover, *R* showed a correlation with the sensitivity of Tar compounds. These results showed that the affinity of the aspartate analogs for Tar could be determined by using the distance between the carboxyl groups. Only the oxaloacetate could not be classified using the distance dependency of the carboxyl groups. Recently, Bi reported antagonist of Tar, which binds to the periplasmic domain of Tar but does not act as an attractant (Bi et al., 2013). The antagonist of the Tar reported in the previous study did not form hydrogen bonds between a donor group in the attractant and the main-chain carbonyls (Y149 and/or Q152); this interaction was suggested to trigger the signal transduction of Tar. The oxaloacetate had the second largest *q*^−^ in our datasets (–224 kJ/mol). Therefore, this compound might fail to form the hydrogen bond between the residues on α4 helix; oxaloacetate might be antagonist of the Tar. The quantification of the *q*^−^ might provide the clue to predict the antagonist of the Tar. In addition to the distance dependency, we demonstrated that the amino group was not essential for classification as an attractant of Tar. Several residues of the α4 helix of Tar have been reported as binding the α amino group on l-aspartate (Tyr-149, Phe-150, Gln-152, and Thr-154; Tajima et al., 2011). Our result suggested that the interaction between α4 helix and amino groups was not essential for ligand recognition by Tar. Instead, it improves the binding affinity of the compound. Nonetheless, this result narrowed down the possible residues essential for detecting attractants of Tar, since only Arg-64 on the α1 helix was left. Arg-64 is known to make a hydration bond with the α carboxyl group of l-aspartate. For β carboxyl group of the l-aspartate, Ser-68 of α1 helix and Arg-69′, Arg-73′ of the antiparallel α1 helix have been reported (Tajima et al., 2011). In this report, Arg-73′ was essential for recognition of l-aspartate, but Ser-68 was not essential (Tajima et al., 2011). Given these results, the residue essential to ligand recognition by Tar could be narrowed down to three arginine residues, which were known as the arginine triplet (Arg-64, Arg-69′, and Arg-73′). Arg-64 of Tar is known to form a hydrogen bond with the α carboxyl group on l-aspartate, and Arg-69′ and Arg-73′ with the β carboxyl group on l-aspartate. Therefore, these essential residues can be crosslinked by two carboxyl groups on l-aspartate (Figure 6B). The distance *R* should affect the distance between carboxyl groups and the arginine residue. This negative correlation between the sensitivities and *R* might be affected by the electrostatic interaction between carboxyl groups and the arginine residue.

From these results, we propose the following model as a common mechanism for ligand recognition by Tar: arginine residues on the α1 helix (Arg-64) and antiparallel α1 helix (Arg-69′ and Arg-73′) are crosslinked by compounds with strong negative charges on both poles (Figure 6B). Moreover, the strength of the crosslink formation is determined by the distance between the carboxyl groups (*R*) (Figure 6C). Such rules for ligand recognition can be utilized for drug discovery, including targets of GPCRs. Therefore, finding such rules might provide efficient strategies for drug design. However, some limitations are worth noting. This model can only be applied to compounds with two carboxyl groups. In a future study, we would like to clarify whether this rule can be applied to residues with any functional groups other than the carboxyl groups. Moreover, the binding of compounds to Tar was not experimentally validated in this study. Future work should quantify the binding affinity of Tar by measuring the *K*_D_ of the purified binding fragment of Tar using ITC.

In summary, the attractants and non-attractants for Tar and Tsr could be classified with only descriptors with a single threshold each: *q*^−^ = 280 kJ/mol and *R* ~ 4 Å. For compounds carrying two-carboxyl groups, 16/17 compounds were correctly classified by assuming the *R* of the attractant to be <4 Å. The remaining compounds could be classified with a high accuracy (33/36) by assuming *q*^−^ of attractant to be <280 kJ/mol. These results showed that 92% (49/53) of ligands of Tar and Tsr can be predicted by using only two descriptors. Moreover, each descriptor was related to the respective ligands for Tar (*R*) and Tsr (*q*^−^). The relation between *R* and sensitivity of binding to Tar highlighted the importance of the arginine triplet of the ligand binding pocket. The amino groups were not essential for ligand recognitions by Tar. On the other hand, the descriptor *q*^−^ reinforced the importance of amino groups for ligand recognition by Tsr. We propose that the selective importance of the amino groups could explain the differential ligand specificity to Tar and Tsr, which are highly homologous (Tajima et al., 2011).

## Author Contributions

The study was conceived by TS. TS and RM acquired the data. TS, YY, YN, and MO designed the data analysis TS and YN interpreted the data. Data analysis: TS and RM. Writing of the manuscript: TS and HK.

## Conflict of Interest Statement

The authors declare that the research was conducted in the absence of any commercial or financial relationships that could be construed as a potential conflict of interest.
