# Supplementary material for: Logistic Regression of Ligands of Chemotaxis Receptors Offers Clues about Their Recognition by Bacteria
Source: Front Bioeng Biotechnol. 2018 Jan 22;5:88. doi: 10.3389/fbioe.2017.00088 (PMC5786873; doi:10.3389/fbioe.2017.00088)
Supplement: Supplementary file 1 [file Table_1.docx]

**Table S1. Physicochemical properties of compounds**

| No. | Name of compounds | *K*_D_ | Formula | Descriptors | | | | | | | | | | | | | | | | | | | | Number of  functional groups | | |
| --- | --- | --- | --- | --- | --- | --- | --- | --- | --- | --- | --- | --- | --- | --- | --- | --- | --- | --- | --- | --- | --- | --- | --- | --- | --- | --- |
|  |  |  |  | *E* | *E*_aq_ | *E*_sol_ | *M*_W_ | *E*_H_ | *E*_L_ | *E*_H_-*E*_L_ | *D* | *A*_CPK_ | *PSA* | *V*_CPK_ | *O*_CPK_ | *AA* | *PA* | *q*^–^ | *APA* | *q*^ion–^ | *q*^+^ | *LogP* | *P* | *N*_Carboxyl_ | *N*_Amino_ | *N*_Hydroxyl_ |
|  |  |  |  | kJ/mol | kJ/mol | kJ/mol | Da | eV | eV | eV | debye | Å^2^ | Å^2^ | Å^3^ | - | Å^2^ | Å^2^ | kJ/mol | Å^2^ | kJ/mol | kJ/mol | - | - |  |  |  |
| 1 | Acetate |  | C_2_H_4_O_2_ | -423 | -464 | -41 | 60 | -11.4 | 0.5 | -11.9 | 2.1 | 85 | 35 | 62 | 1.12 | 69 | 40 | -260 | 36 | 54 | 178 | -0.3 | 44 | 1 |  |  |
| 2 | N-acetyl-L-aspartate | 1E-03 | C_6_H_9_NO_5_ | -936 | -1028 | -93 | 175 | -10.5 | -0.4 | -10.1 | 4.0 | 191 | 92 | 159 | 1.35 | 121 | 100 | -306 | 72 | 52 | 222 | -1.7 | 52 | 2 |  |  |
| 3 | N-acetyl-L-serine | 5E-05 | C_5_H_9_NO_4_ | -718 | -799 | -81 | 147 | -10.8 | -0.2 | -10.5 | 2.7 | 169 | 77 | 139 | 1.3 | 109 | 97 | -281 | 68 | 48 | 216 | -1.8 | 50 | 1 |  | 1 |
| 4 | β-Alanine |  | C_3_H_7_NO_2_ | -398 | -463 | -65 | 89 | -10.0 | 0.3 | -10.2 | 3.0 | 118 | 60 | 90 | 1.21 | 85 | 63 | -322 | 51 | 55 | 189 | -1.1 | 46 | 1 | 1 |  |
| 5 | L-α-Aminoadipate |  | C_6_H_11_NO_4_ | -795 | -877 | -81 | 161 | -10.4 | -0.1 | -10.3 | 2.7 | 190 | 94 | 156 | 1.36 | 117 | 88 | -292 | 66 | 53 | 198 | -1.0 | 52 | 2 | 1 |  |
| 6 | L-α-Amino-n-butyrate | 3E-04 | C_4_H_9_NO_2_ | -412 | -455 | -43 | 103 | -10.1 | 0.1 | -10.2 | 1.0 | 137 | 58 | 109 | 1.24 | 94 | 45 | -315 | 32 | 55 | 194 | -0.4 | 48 | 1 | 1 |  |
| 7 | β-Amino-n-butyrate | 3E-02 | C_4_H_9_NO_2_ | -433 | -485 | -52 | 103 | -9.7 | 0.2 | -9.9 | 3.0 | 136 | 58 | 109 | 1.24 | 94 | 52 | -346 | 38 | 53 | 188 | -0.8 | 48 | 1 | 1 |  |
| 8 | γ-Amino-n-butyrate |  | C_4_H_9_NO_2_ | -419 | -485 | -66 | 103 | -9.7 | 0.3 | -10.1 | 1.3 | 138 | 60 | 109 | 1.25 | 96 | 56 | -330 | 44 | 55 | 187 | -0.8 | 48 | 1 | 1 |  |
| 9 | 2-Aminoethanol | 1E-03 | C_2_H_7_NO | -203 | -269 | -66 | 61 | -9.7 | 2.3 | -12.0 | 1.7 | 96 | 46 | 70 | 1.17 | 74 | 49 | -325 | 38 | 47 | 171 | -1.2 | 44 |  | 1 | 1 |
| 10 | α-Aminoisobutyrate | 2E-05 | C_4_H_9_NO_2_ | -431 | -476 | -45 | 103 | -10.2 | 0.2 | -10.4 | 1.7 | 136 | 59 | 109 | 1.24 | 90 | 37 | -322 | 25 | 50 | 183 | -0.5 | 48 | 1 | 1 |  |
| 11 | DL-2-Amino-1-propanol | 3E-03 | C_3_H_9_NO | -225 | -277 | -52 | 75 | -9.6 | 2.5 | -12.1 | 1.1 | 114 | 44 | 88 | 1.19 | 82 | 40 | -332 | 27 | 46 | 202 | -0.9 | 46 |  | 1 | 1 |
| 12 | Ammonia | 2E-04 | H_3_N | -12 | -61 | -49 | 17 | -10.2 | 4.4 | -14.6 | 2.4 | 42 | 42 | 23 | 1.07 | 42 | 29 | -359 | 29 | 46 | 131 |  | 40 |  | 1 |  |
| 13 | D-Aspartate | 1E-05 | C_4_H_7_NO_4_ | -728 | -817 | -89 | 133 | -10.5 | 0.0 | -10.5 | 4.4 | 147 | 91 | 118 | 1.26 | 95 | 85 | -293 | 64 | 55 | 197 | -1.7 | 49 | 2 | 1 |  |
| 14 | L-Aspartate | 6E-08 | C_4_H_7_NO_4_ | -737 | -827 | -91 | 133 | -10.5 | -0.1 | -10.4 | 3.1 | 147 | 93 | 118 | 1.26 | 95 | 85 | -287 | 66 | 55 | 191 | -1.7 | 49 | 2 | 1 |  |
| 15 | L-aspartate diamide | 1E-03 | C_4_H_9_N_3_O_2_ | -407 | -482 | -75 | 131 | -9.9 | 0.5 | -10.4 | 3.5 | 154 | 98 | 125 | 1.27 | 102 | 80 | -307 | 64 | 52 | 162 | -3.0 | 49 |  | 3 |  |
| 16 | DL-Aspartate dimethyl ester | 1E-03 | C_6_H_11_NO_4_ | -717 | -781 | -65 | 161 | -10.2 | 0.2 | -10.4 | 2.7 | 193 | 67 | 159 | 1.36 | 125 | 89 | -309 | 64 | 56 | 161 | -1.2 | 52 |  | 1 |  |
| 17 | L-Aspartate α-ethyl ester | 5E-06 | C_6_H_11_NO_4_ | -795 | -858 | -63 | 161 | -10.0 | 0.3 | -10.3 | 2.0 | 189 | 75 | 158 | 1.33 | 124 | 67 | -289 | 48 | 53 | 190 | -1.1 | 52 | 1 | 1 |  |
| 18 | L-Aspartate β-methyl ester | 5E-06 | C_5_H_9_NO_4_ | -728 | -804 | -76 | 147 | -10.4 | 0.0 | -10.4 | 2.6 | 171 | 81 | 139 | 1.32 | 111 | 89 | -299 | 67 | 55 | 190 | -1.4 | 50 | 1 | 1 |  |
| 19 | L-Citrullin | 2E-04 | C_6_H_13_N_3_O_3_ | -555 | -654 | -99 | 175 | -10.2 | 0.1 | -10.3 | 3.0 | 208 | 108 | 170 | 1.4 | 132 | 104 | -308 | 77 | 52 | 189 | -2.0 | 53 | 1 | 2 |  |
| 20 | L-Cysteate | 1E-04 | C_3_H_7_NO_5_S | -866 | -998 | -133 | 169 | -10.5 | -0.5 | -10.0 | 4.1 | 164 | 116 | 130 | 1.32 | 98 | 106 | -307 | 76 | 53 | 321 | -2.0 | 50 | 1 | 1 |  |
| 21 | Formate |  | CH_2_O_2_ | -356 | -396 | -40 | 46 | -11.5 | 0.1 | -11.6 | 1.6 | 64 | 35 | 43 | 1.07 | 54 | 40 | -247 | 39 | 56 | 190 | -0.6 | 42 | 1 |  |  |
| 22 | Fumarate | 3E-04 | C_4_H_4_O_4_ | -642 | -702 | -60 | 116 | -11.7 | -1.7 | -10.0 | 0.0 | 130 | 69 | 103 | 1.23 | 94 | 55 | -226 | 47 | 56 | 204 | 0.0 | 47 | 2 |  |  |
| 23 | DL-Glycerate |  | C_3_H_6_O_4_ | -714 | -788 | -74 | 106 | -11.0 | -0.2 | -10.9 | 2.0 | 122 | 73 | 95 | 1.21 | 82 | 68 | -265 | 53 | 46 | 206 | -1.4 | 47 | 1 |  | 2 |
| 24 | L-Homocystein |  | C_4_H_9_NO_2_S | -383 | -440 | -57 | 135 | -9.1 | -0.1 | -9.0 | 2.4 | 158 | 58 | 127 | 1.29 | 106 | 69 | -284 | 54 | 44 | 204 | -0.6 | 50 | 1 | 1 |  |
| 25 | L-Homoserine | 1E-04 | C_4_H_9_NO_3_ | -570 | -646 | -76 | 119 | -10.3 | 0.0 | -10.3 | 2.4 | 146 | 78 | 117 | 1.27 | 96 | 68 | -283 | 49 | 46 | 202 | -1.5 | 48 | 1 | 1 | 1 |
| 26 | DL-erythro-β-hydroxyaspartate | 5E-04 | C_4_H_7_NO_5_ | -921 | -1015 | -94 | 149 | -10.4 | -0.4 | -9.9 | 2.9 | 158 | 110 | 127 | 1.29 | 98 | 84 | -284 | 62 | 49 | 228 | -2.3 | 49 | 2 | 1 | 1 |
| 27 | DL-threo-β-Hydroxyaspartate | 1E-04 | C_4_H_7_NO_5_ | -873 | -965 | -92 | 149 | -11.0 | -0.5 | -10.5 | 3.2 | 155 | 109 | 126 | 1.28 | 97 | 86 | -247 | 64 | 48 | 225 | -2.3 | 49 | 2 | 1 | 1 |
| 28 | Hydroxypyruvate |  | C_3_H_4_O_4_ | -630 | -694 | -64 | 104 | -10.9 | -1.7 | -9.2 | 1.8 | 116 | 68 | 90 | 1.19 | 85 | 68 | -223 | 58 | 46 | 225 | -0.7 | 47 | 1 |  | 1 |
| 29 | Isoasparagine | 5E-04 | C_4_H_8_N_2_O_3_ | -559 | -648 | -89 | 132 | -10.1 | 0.3 | -10.4 | 2.3 | 152 | 98 | 122 | 1.28 | 99 | 83 | -289 | 62 | 54 | 193 | -2.3 | 49 | 1 | 2 |  |
| 30 | Isoserine | 5E-04 | C_3_H_7_NO_3_ | -546 | -618 | -72 | 105 | -10.1 | -0.3 | -9.8 | 3.0 | 125 | 77 | 98 | 1.22 | 87 | 62 | -324 | 48 | 53 | 215 | -1.8 | 47 | 1 | 1 | 1 |
| 31 | α-Ketoglutarate |  | C_5_H_6_O_5_ | -857 | -928 | -71 | 146 | -10.9 | -1.4 | -9.5 | 0.7 | 159 | 83 | 129 | 1.29 | 105 | 91 | -244 | 75 | 55 | 212 | -0.4 | 50 | 2 |  |  |
| 32 | DL-Lactate |  | C_3_H_6_O_3_ | -578 | -627 | -49 | 90 | -10.9 | -0.1 | -10.8 | 1.0 | 112 | 52 | 87 | 1.18 | 80 | 45 | -245 | 35 | 50 | 201 | -0.5 | 46 | 1 |  | 1 |
| 33 | L-Malate | 6E-04 | C_4_H_6_O_5_ | -926 | -1005 | -79 | 134 | -11.2 | -0.3 | -10.9 | 1.1 | 143 | 86 | 115 | 1.25 | 93 | 74 | -243 | 59 | 55 | 210 | -1.3 | 48 | 2 |  | 1 |
| 34 | Methylammonium | 2E-04 | CH_5_N | -4 | -44 | -40 | 31 | -9.4 | 3.6 | -13.0 | 2.1 | 66 | 26 | 44 | 1.09 | 60 | 20 | -358 | 19 | 49 | 108 | -0.7 | 42 |  | 1 |  |
| 35 | DL-α-Methylaspartate | 5E-07 | C_5_H_9_NO_4_ | -769 | -840 | -70 | 147 | -10.4 | -0.2 | -10.2 | 2.7 | 164 | 92 | 136 | 1.28 | 100 | 79 | -292 | 55 | 55 | 202 | -1.3 | 50 | 2 | 1 |  |
| 36 | DL-β-Methylaspartate | 3E-04 | C_5_H_9_NO_4_ | -767 | -853 | -85 | 147 | -10.2 | -0.3 | -9.9 | 4.4 | 160 | 86 | 136 | 1.25 | 104 | 97 | -269 | 77 | 50 | 212 | -1.1 | 50 | 2 | 1 |  |
| 37 | N-Methyl-DL-aspartate | 1E-03 | C_5_H_9_NO_4_ | -763 | -857 | -94 | 147 | -9.7 | -0.3 | -9.4 | 4.0 | 165 | 73 | 139 | 1.27 | 108 | 81 | -283 | 65 | 53 | 226 | -1.2 | 50 | 2 |  |  |
| 38 | DL-α-Methylglutamate |  | C_6_H_11_NO_4_ | -795 | -873 | -78 | 161 | -10.1 | 0.1 | -10.2 | 1.2 | 186 | 92 | 155 | 1.33 | 113 | 84 | -318 | 58 | 53 | 196 | -1.0 | 52 | 2 | 1 |  |
| 39 | DL-α-Methylserine | 2E-04 | C_4_H_9_NO_3_ | -590 | -655 | -65 | 119 | -10.2 | 0.0 | -10.2 | 2.9 | 142 | 74 | 116 | 1.23 | 92 | 69 | -313 | 48 | 46 | 190 | -1.4 | 48 | 1 | 1 | 1 |
| 40 | N-methyl-L-serine | 3E-04 | C_4_H_9_NO_3_ | -544 | -625 | -81 | 119 | -9.6 | 0.0 | -9.7 | 2.1 | 146 | 66 | 118 | 1.25 | 97 | 64 | -318 | 47 | 48 | 198 | -1.2 | 49 | 1 |  | 1 |
| 41 | O-methyl-DL-serine | 2E-04 | C_4_H_9_NO_3_ | -529 | -591 | -62 | 119 | -10.1 | 0.0 | -10.2 | 3.9 | 148 | 65 | 118 | 1.27 | 104 | 78 | -327 | 60 | 50 | 190 | -1.4 | 49 | 1 | 1 |  |
| 42 | 2-Methylsuccinate | 5E-03 | C_5_H_8_O_4_ | -802 | -861 | -59 | 132 | -11.4 | 0.1 | -11.5 | 0.5 | 154 | 69 | 126 | 1.27 | 100 | 60 | -248 | 46 | 53 | 192 | -0.1 | 49 | 2 |  |  |
| 43 | L-Ornithine | 2E-03 | C_5_H_12_N_2_O_2_ | -403 | -487 | -84 | 132 | -9.8 | 0.1 | -9.9 | 2.0 | 170 | 83 | 138 | 1.32 | 111 | 79 | -325 | 59 | 53 | 188 | -1.6 | 50 | 1 | 2 |  |
| 44 | Oxaloacetate |  | C_4_H_4_O_5_ | -827 | -902 | -75 | 132 | -11.0 | -1.4 | -9.6 | 1.8 | 137 | 83 | 110 | 1.23 | 92 | 84 | -224 | 71 | 56 | 210 | -0.3 | 48 | 2 |  |  |
| 45 | Propionate |  | C_3_H_6_O_2_ | -426 | -461 | -35 | 74 | -11.2 | 0.4 | -11.6 | 2.1 | 104 | 35 | 80 | 1.16 | 78 | 29 | -273 | 23 | 54 | 176 | 0.4 | 45 | 1 |  |  |
| 46 | Pyruvate |  | C_3_H_4_O_3_ | -490 | -534 | -44 | 88 | -10.7 | -1.2 | -9.5 | 1.3 | 108 | 49 | 83 | 1.17 | 81 | 59 | -224 | 50 | 55 | 204 | 0.0 | 46 | 1 |  |  |
| 47 | D-Serine | 3E-05 | C_3_H_7_NO_3_ | -541 | -620 | -79 | 105 | -10.2 | -0.1 | -10.2 | 3.5 | 126 | 77 | 98 | 1.22 | 85 | 70 | -323 | 53 | 49 | 208 | -1.8 | 47 | 1 | 1 | 1 |
| 48 | L-Serine | 3E-07 | C_3_H_7_NO_3_ | -551 | -630 | -79 | 105 | -10.3 | -0.1 | -10.2 | 1.1 | 125 | 77 | 98 | 1.22 | 86 | 71 | -310 | 55 | 49 | 201 | -1.8 | 47 | 1 | 1 | 1 |
| 49 | L-Serine amide | 1E-05 | C_3_H_8_N_2_O_2_ | -378 | -452 | -74 | 104 | -10.4 | 0.6 | -11.0 | 2.2 | 130 | 82 | 101 | 1.24 | 89 | 68 | -294 | 51 | 49 | 192 | -2.4 | 47 |  | 2 | 1 |
| 50 | L-Serine methyl ester | 1E-05 | C_4_H_9_NO_3_ | -542 | -611 | -69 | 119 | -10.1 | 0.2 | -10.2 | 3.2 | 148 | 64 | 119 | 1.27 | 104 | 71 | -321 | 55 | 50 | 187 | -1.5 | 49 |  | 1 | 1 |
| 51 | DL-Serinol |  | C_3_H_9_NO_2_ | -395 | -468 | -73 | 91 | -10.2 | 2.0 | -12.1 | 2.4 | 123 | 62 | 96 | 1.21 | 85 | 69 | -280 | 49 | 44 | 192 | -1.7 | 46 |  | 1 | 2 |
| 52 | Succinate | 2E-04 | C_4_H_6_O_4_ | -780 | -845 | -64 | 118 | -11.6 | 0.1 | -11.7 | 0.0 | 135 | 69 | 107 | 1.24 | 80 | 83 | -246 | 60 | 17 | 187 | -0.6 | 47 | 2 |  |  |
| 53 | Glutamic acid | 5E-06 | C_5_H_9_NO_4_ | -761 | -843 | -82 | 147 | -10.4 | -0.1 | -10.3 | 3.0 | 164 | 90 | 136 | 1.28 | 102 | 84 | -285 | 63 | 53 | 202 | -1.4 | 50 | 2 | 1 |  |

No. shows serial number of compounds. *K*_D_ (M) shows apparent dissociation constant to induce attractant response of WT *E. coli* cell (Mesibov and Adler, 1972), and the blank showed non-attractant. Definition of each descriptors were summarized in Materials and Methods. *N*_Carboxyl_, *N*_Amino_, and *N*_Hydroxyl_ shows number of carboxyl, amino, and hydroxyl groups on each compound. Hydroxyl groups on carboxyl group was not counted as *N*_Hydroxyl_.
